# Pharmacokinetics of cannabidiol and its two main phase I metabolites in Connemara ponies

**DOI:** 10.3389/fvets.2025.1599934

**Published:** 2025-06-27

**Authors:** Kata Wermer, Orsolya Korbacska-Kutasi, Róbert Berkecz, Dezső Csupor, Nóra Ágh, Anita Sztojkov-Ivanov, Dániel Cserhalmi

**Affiliations:** ^1^Department of Botany, University of Veterinary Medicine Budapest, Budapest, Hungary; ^2^Department of Animal Nutrition and Clinical Dietetics, University of Veterinary Medicine Budapest, Budapest, Hungary; ^3^Institute of Pharmaceutical Analysis, Faculty of Pharmacy, University of Szeged, Szeged, Hungary; ^4^Department of Forensic Medicine, Albert Szent-Györgyi Health Centre, Szeged, Hungary; ^5^Faculty of Pharmacy, Institute of Clinical Pharmacy, University of Szeged, Szeged, Hungary; ^6^Institute for Translational Medicine, Medical School, University of Pécs, Pécs, Hungary; ^7^HUN-REN-PE Evolutionary Ecology Research Group, University of Pannonia, Veszprém, Hungary; ^8^Behavioral Ecology Research Group, Center for Natural Sciences, University of Pannonia, Veszprém, Hungary; ^9^Institute of Pharmacodynamics and Biopharmacy, Faculty of Pharmacy, University of Szeged, Szeged, Hungary

**Keywords:** cannabidiol, horse, equine, CBD metabolism, pharmacokinetics, cannabinoid

## Abstract

**Introduction:**

Cannabidiol (CBD) has shown potential therapeutic benefits in veterinary medicine, but further investigations are needed to establish its pharmacokinetics and therapeutic dosing in horses and ponies. The present study aimed to investigate the pharmacokinetic properties of CBD in Connemara ponies following oral administration of CBD oil.

**Methods:**

Ten healthy ponies received a single oral dose of CBD oil at 2 mg/kg. Blood samples were collected before dosing and up to 16 days post-administration, with physical examinations conducted at baseline and at 8, 12, and 24 h post-dose. Additional blood samples were taken at baseline and 24 h for hematological and biochemical analyses. Targeted UHPLC–MS/HRMS measurements quantified CBD and its metabolites, 7-hydroxy-CBD (7-OH-CBD) and 7-carboxy-CBD (7-COOH-CBD).

**Results:**

The CBD oil was well-tolerated, with no side effects. A significant decrease in heart rate was observed after 24 h. Changes in hematological and biochemical markers included elevated urea, slight increases in chloride, Gamma-glutamyl transferase, Total bilirubin, Lactate dehydrogenase, and a minor reduction in WBC count. CBD was detectable in 4 ponies on day 7, but none on day 12. The 7-COOH-CBD metabolite remained detectable up to day 16 in all subjects. The pharmacokinetic parameters for CBD were C_max_ = 31.472 ± 8.080 ng/mL, t_max_ = 2.111 ± 0.928 h, t_1/2_ = 12.563 ± 3.054 h, and Vz/*F* = 198.757 ± 49.123 L/kg.

**Discussion:**

The pharmacokinetic characteristics of CBD observed in the present study are consistent with previous research in warmblood horses and provide a foundation for future studies to evaluate the therapeutic efficacy and long-term safety of CBD in ponies.

## Introduction

Cannabidiol (CBD) has rapidly gained huge popularity as an alternative or adjunct treatment in human and veterinary medicine as well. CBD is one of the many cannabinoids derived from the *Cannabis sativa* L. plant. Although it is psychoactive, it is non-intoxicating—unlike tetrahydrocannabinol (THC)—which makes it an excellent candidate for medical research (1, 2). The mechanism of action is associated with the endocannabinoid system (ECS), which is involved in the homeostasis of the organism, including neuromodulatory functions in the central nervous system (3). Initially, the ECS was defined by cannabinoid receptors CB1 and CB2, endogenous cannabinoids, and the enzymes responsible for their synthesis and degradation (4). However, this simplified view has evolved into the broader concept of the “endocannabinoidome”, encompassing a complex network of additional cannabinoid-related receptors, such as nuclear peroxisome proliferator-activated (PPARs), serotonin (5-HT), opioid, G protein-coupled receptors (GPRs) and transient receptor potential (TRPs) channels (1, 5, 6), and endocannabinoid-like molecules (7). This expanded system is now recognized as a highly intricate signaling network with numerous overlapping pathways (7).

CBD’s therapeutic potential arises from its diverse mechanisms of action, although the precise pathways remain under active investigation (8). CB1 is predominantly expressed in the central and peripheral nervous systems, particularly in brain regions related to motor control, emotion, and energy regulation, but is also present in peripheral tissues such as vascular endothelium, synovium, skeletal muscle, adipose tissue, and the reproductive system (3, 6, 9, 10). CB2, while primarily associated with the immune system, is broadly distributed across various peripheral tissues, including epithelial cells, fibroblasts, endothelial cells, and synovial cells (3, 6, 9, 10).

Cannabinoid and cannabinoid-related receptors have also been identified in humans and other mammals, such as dogs and horses. Zamith Cunha et al. (11) revealed the expression of cannabinoid and cannabinoid-related receptors (CB1, CB2, GPR55, PPARγ, and TRPV1) in the trigeminal ganglia neurons and satellite glial cells of horses, suggesting their potential role in modulating trigeminal nerve function and neuropathic pain. In another study, Galiazzo et al. found (5) Cannabinoid-related receptors (TRPV1, PPARγ, GPR55, and GPR3) in sensory neurons, satellite glial cells (SGCs), macrophages, and other interneuron cells of the equine dorsal root ganglion (DRG). These findings provide an anatomical foundation for further research into the potential therapeutic use of non-psychotropic cannabinoid agonists in pain management for horses. Galiazzo et al. (12) also investigated the localization of cannabinoid and cannabinoid-related receptors in the horse ileum, finding immunoreactivity for CB1, CB2, and 5-HT1aR in epithelial cells, CB2 and 5-HT1aR in lamina propria inflammatory cells, and CB1, TRPA1, and PPARα in enteric neurons. Additionally, CB1 and PPARα were identified in enteric glial cells, while PPARα was present in smooth muscle cells of the tunica muscularis and blood vessels. These findings underscore the significant role of the endocannabinoid system in gut homeostasis. Kupczyk et al. (13) presented detailed gene and protein expression profiles of CB1 and CB2 receptors in whole equine skin tissue and primary cell types such as keratinocytes, fibroblasts, and other dermal cells. Their findings support the development of peripheral cannabinoid-based therapies for clinical dermatology in equine veterinary medicine. It’s also worth mentioning that the distribution of receptors can vary among species. For instance, more cannabinoid receptors are expressed in the dog’s brain than in humans, making dogs generally more sensitive to cannabinoids (14).

CBD’s major and most well-studied metabolites are 7-hydroxy-CBD (7-OH-CBD), and 7-carboxy-CBD (7-COOH-CBD) (15). The main cannabinoid metabolism takes place in the liver. THC and CBD, undergo hydroxylation or oxidation by cytochrome P450 (CYP450) enzymes, followed by glucuronidation via UDP glucuronosyltransferase (UGT) enzymes (15).

Several beneficial effects have been attributed to CBD, including antipsychotic, antidepressant, anxiolytic, anticonvulsant, antidiabetic, analgesic, antioxidant, and anti-inflammatory properties (6, 16, 17). In human medicine, CBD can be a part of the management of epilepsy and multiple sclerosis. In veterinary medicine, CBD has been mostly studied in cases of osteoarthritis, neuropathies, postoperative pain management, epilepsy, anxiety, and allergic and respiratory diseases. Its efficacy has primarily been investigated in laboratory animals (such as rats, mice, and guinea pigs) as well as in dogs (18–24).

The potential targets of CBD in equine medicine are pain management related to osteoarthritis and laminitis, treatment of stereotypic behavior issues and stress relief (25–29). The effectiveness of CBD is still not proven, the scientific or even the empirical evidence is inconsistent or lacking completely and the use of CBD products are rather anecdotal (30). Other concerns related to the use of CBD are possible adverse effects, like liver toxicity, drug–drug interactions, adequate regulatory oversight of retail CBD products (31).

In equine medicine, the vast majority of publications are focusing on the pharmacokinetics of CBD. Previous experiments used different types of CBD products, which can be one explanation to the diversity of the results (30). Some studies include co-administration of THC (32–35), which has similarities to CBD (structure and lipophilicity) and could influence its pharmacokinetics (1). This suggest that additional research is needed with particular care given to the chosen product, the method of administration and the doses.

Oral CBD doses in horses typically range from 0.1 to 3 mg/kg, with reported plasma concentrations generally remaining below 20 ng/mL (32, 36–45). Based on studies so far, it appears that the pharmacokinetics of CBD in horses are more similar to humans than dogs (27). By administering similar doses of CBD orally, a much higher peak plasma concentration can be achieved in dogs. In one report, the median maximal concentration of CBD was 102.3 ng/mL and 590.8 ng/mL, respectively, with 2 and 8 mg/kg oral doses (35).

Effective plasma concentrations have not yet been established for horses. It may highly depend on the species and the desired therapeutic effect. In humans, therapeutic effects have been reported with plasma concentrations ranging from 5–10 to 15–30 ng/mL after oral CBD doses of 2.5–10 mg/kg (3). In one study, 300 mg of oral CBD was found to be anxiolytic in healthy people who were giving public speeches. Interestingly, CBD-induced anxiolytic effects showed an inverted U-shaped dose–response curve, with anxiety reduced in 300 mg CBD but not in the 100 and 900 mg CBD groups (46).

Even though the knowledge of CBD pharmacokinetics, metabolism and also controlled evidence for the therapeutic efficacy in several health conditions is incomplete, there is a noticeable increase in the use of CBD products among horse owners. In one survey, more than half of respondents (67.2%) believed that products containing CBD were beneficial for horses (47). There are several types of CBD products available for horse owners, however only a handful of them passed quality control inspections. This fact indicates that the manufacturers do not verify whether the promised CBD concentration is present (48).

Based on studies conducted so far, further investigation of CBD pharmacokinetics is necessary to establish appropriate therapeutic doses and plasma concentrations in horses and ponies. The aim of this study was to investigate the pharmacokinetic properties of CBD in Connemara ponies following oral administration of a CBD oil. Based on the findings, the authors plan to conduct further efficacy studies and to gain a more accurate understanding of the elimination of CBD and its two main metabolites (7-OH-CBD and 7-COOH-CBD). The primary hypothesis was that oral administration of 2 mg/kg CBD oil would result in measurable plasma concentrations of the parent compound and its two main phase I metabolites, with pharmacokinetic characteristics that could inform future research in ponies.

## Materials and methods

### Animals

Ten healthy Connemara ponies (6 non-pregnant mares and 4 geldings, weight: 488.5 ± 20.8, age: 5–25 years) were included in the study. All ponies were housed on the same ranch, fed hay from hay nets, and had free access to water and salt. At night, the horses were kept in individual boxes with straw bedding and had access to pasture for 8 h during the day. Animals were considered healthy based on clinical history and physical examination, and none had received any medications for at least 6 weeks prior to the start of the study. The inclusion criteria required that the horses be Connemara ponies, housed on the same ranch, and show no signs of illness based on a basic physical examination. All subjects met these criteria.

### CBD product

The used CBD product was a commercially available CBD oil (Pure CBD Oil 10 BD MCT, Pure Production AG, Etzmatt 273, CH-4314 Zeiningen) containing natural hemp extract with medium-chain triglyceride (MCT) coconut oil with no THC content. The content was verified by the laboratory of Pure Production AG.

### Animal treatment

The determination of oral CBD dose was according to previously published studies. The most common dose used in horses was between 0.1 to 3 mg/kg and CBD was detectable in equine plasma even with lower doses, thus 2 mg/kg oral CBD dose was applied (32, 36–45).

The horses were physically examined and their body weight was estimated using a weight tape the day before dosing. Based on this weight, a CBD dose of 2 mg/kg was calculated, and 8.3–10.7 mL of oil was administered orally using a dosing syringe.

The last feeding of hay occurred 12 h prior to CBD administration, and hay was withheld for an additional 30 min following the administration of the CBD oil; however, the horses had access to straw bedding, which allowed for some foraging, so the fasting period was not absolute. On the first day, the horses were kept in boxes with straw bedding, and had access to pasture after the 6th sample collection for 4 h.

Physical examination was performed prior (baseline) and at 8, 12, 24 h following oil administration. The examination included the record of heart rate, respiratory rate, temperature, bowel movements in all four quadrants [absent (0), physiological (1), slightly increased motility (2), hypermotility (3)] and also ataxia degree, auditory (hand clap 60 cm from head) and touch (running hand from ears to shoulder) stimulation response scoring. A 4 point numerical scale was used for the assessment of ataxia [no change from normal non sedated state (0); stable but swaying (1); swaying and leaning against the wall (2); swaying, leaning on the wall with carpi flexed and/or hind limbs crossed (3)] (33). The auditory and touch stimuli responses were also scored [no reaction (0), slight head movement with no limb movement (1), vigorous head movement with no limb movement (2), vigorous head movement with movement of one or more limb (3)] (33).

The horses were closely monitored throughout the experiment for side effects including brady- or tachycardia, severe signs of hypotension, depression, hypersalivation, hypothermia, hyperesthesia, diarrhea, colic, urinary incontinence, mydriasis, incoordination, ataxia and seizures (49, 50).

### Blood sampling for CBD and metabolites analyses

Blood samples (9 mL) were collected via direct venipuncture (needle 22 G) into vacuette® LH lithium heparin blood tubes (Greiner Bio-One GmbH, Kremsmünster, Austria) at time 0 (immediately before administration), 30 min, and 1, 2, 3, 4, 6, 8, 12, 24, 48, 72, 168, 288, 336, and 384 h following administration. Samples were stored at room temperature approximately 60 min until centrifugation (3,200 RPM, 10 min). The plasma (4 mL) was transferred to cryovials and stored at −20°C until analysis.

### Blood hematology and biochemistry

Blood samples were harvested from the jugular vein into K3-EDTA tubes (Sarstedt Nümbrecht, Germany) for complete blood count (CBC), and into plain serum tubes (Sarstedt Nümbrecht, Germany) for biochemical profile before and 24 h after the oil administration. Samples were analyzed by a commercial laboratory (PraxisLab Ltd., Budapest, Hungary). CBCs were run on a Siemens, Advia 2,120 hematology analyzer, whilst biochemical tests were run on a Beckman DCX700 analyzer (Beckman Coulter, Brae, CA, USA) using system reagents. Biochemistry panel included total protein, albumin, aspartate aminotransferase (AST), alkaline phosphate (ALP), glutamate dehydrogenase (GLDH), total bilirubin (TBR), gamma-glutamyl transferase (GGT), Creatine kinase (CK), Lactate dehydrogenase (LDH), triglyceride (TG), glucose, creatinine, urea, sodium, potassium, sodium/potassium ratio, chloride, calcium, phosphate.

### CBD and metabolites analysis in horse plasma

#### Sample preparation procedure

For the enrichment of CBD from horse plasma, 10 μL of THC-D3 (100 ng/mL) as internal standard, 10 μL of methanol (for samples) or given calibration solution (for calibration points), 100 μL of saline solution to 400 μL plasma sample and the solution was vortexed thoroughly for 0.5 min. In the next step, 1,000 μL of ethyl-acetate was added, then vortexed and sonicated for 5 min. In the following step, after 10 min centrifugation at 15000 rpm (Universal 320 R, Hettich, Tuttlingen, Germany) at 4°C, the upper layer was collected and evaporated to dryness. The dried extract was reconstituted in 100 μL methanol. Finally, 5 μL aliquots were injected for UHPLC–MS/HRMS analysis.

The following calibration solutions were used to prepare matrix-matched external calibration points: CBD concentrations of 0, 10, 100, 500, 1,000, 2000, and 4,000 ng/mL.

#### Targeted UHPLC–MS/HRMS parameters for quantitation of CBD and its main metabolites

The targeted UHPLC–MS/HRMS measurements were performed on Waters Acquity I-Class UPLC (Milford, MA, UK) connected to Thermo Scientific Orbitrap Q Exactive Plus Hybrid Quadrupole-Orbitrap™ (Waltham, MA, USA) mass spectrometer. The UPLC system was controlled by the MassLynx software (Milford, MA, UK). Data were acquired and evaluated with Xcalibur 4.2. software (Thermo Fisher Scientific, Waltham, MA, USA).

The UHPLC separations were carried out on Kinetex™ XB-C18 column (50 × 2.1 mm, 2.6 μm) with SecurityGuard Ultra C18 cartridge from (4 × 3 mm) from Phenomenex (Torrance, CA, USA). The UHPLC mobile phase A consisted of 1% formic acid aqueous solution and mobile phase B was composed of MeOH with 1% formic acid. The gradient program was 0–0.20 min 20% B, 0.20–1.00 – 3.00 min 20–100 – 100% B, 3.00–3.10 – 4.00 min 100–20 –20% B. The flow rate gradient program was 0–2.00 min 0.3 mL/min, 2.00–2.01 – 3.90 min 0.3–0.5 – 0.5 mL/min, 3.90–3.95 – 4.00 min 0.5–0.3 – 0.3 mL/min. The column temperature was maintained at 50°C; the autosampler temperature was set to 5°C, respectively.

For MS/HRMS detection of targeted compounds, positive heated-electrospray ionization (HESI) was used with the following parameters: capillary temperature 300°C, S-Lens RF level 50, spray voltage 4.0 kV, sheath gas flow 50, spare gas flow 1, and auxiliary gas flow 10 in arbitrary units. In scheduled parallel reaction monitoring with a resolution of 17,500 (FWHM), the AGC setting was defined as 3 × 10^6^ charges and the maximum isolation time was set to 50 ms. The width of the isolation window of the precursor ion was 0.4 Da. The optimized collision energies were the following for the protonated form of CBD (24 eV), 7-OH-CBD (22 eV), 7-COOH-CBD (22 eV) and THC-D3 (24 eV). The effluent was introduced into the HESI source via a 2-position, 6-port divert valve only in 1.3–2.0 min. In the rest of the total run time, the HESI source was rinsed with acetonitrile/water solution (90/10, v/v) at a flow rate of 0.2 mL/min by Waters Acquity I-Class pump (Milford, MA, UK).

#### Pharmacokinetic analysis

Pharmacokinetic parameters after a single oral 2 mg/kg dose of CBD were calculated for the time-concentration profiles using non-compartmental analysis by Phoenix WinNonlin Software, version 8.5.2.4 (Certara Inc., Pennsylvania, USA). The maximum CBD concentration in the plasma (C_max_) and the time of maximum observed concentration (t_max_) were determined from the time versus plasma concentration profiles. The elimination rate constant was calculated as the terminal slope (λ_Z_) by performing linear regression analysis on the terminal phase of the log concentration versus time curve. The area under the zero to 168-h plasma concentration curve (AUC_0-t_) was determined by linear trapezoid rule. The area under the zero to infinity (AUC_0-inf_) was calculated by extrapolation to infinity using the equation AUC_0-inf_ = AUC_0-t_ + C_t_ /λ_Z_, where C_t_ is the concentration measured at 168 h. The elimination half-life (t_½_) of the terminal elimination phase was estimated using the formula t _½_ = 0.693/λ_Z_. Mean residence time (MRT_0- inf_) was calculated with the formula MRT_0- inf =_ AUMC_0- inf_/AUC_0-inf_, where AUMC_0- inf_ is the area under the first moment curve extrapolated to infinity. The oral clearance (Cl/F) and apparent volume of distribution (V_Z_/F) were determined using the equations Cl/F = Dose/AUC_0-inf_ and V_Z_/F = Dose/ (λ_Z_ AUC_0-inf_), respectively, where F is the fraction of dose absorbed. All reported data are means ± standard deviation (SD).

### Statistical analysis

Statistical analyses were performed by R 4.3.3 software (51). To control the kinship between the individuals, father ID as family ID was used in the analysis when it is necessary.

The concentration of CBD was defined in ng/mL, the limit of detection was 0.1 ng/mL. In the case of the two metabolites (7-COOH-CBD, 7-OH-CBD), derived variables were used as peak area ratio and relative standard deviation (%RSD). The limit of detection of peak area ratio was 0.05 in the case of 7-COOH-CBD and 0.03 in 7-OH-CBD (normalized to internal standard). The distribution over time was plotted between the date of administration and after 168 h for all analyzed substances. In the case of CBD, the median, standard error, the maximum plasma concentration (C_max_) and the time to achieve the maximum concentration (T_max_) were calculated in that period.

Non-linear mixed models (nlmer) were built to analyze the temporal variation of the CBD in the plasma with adding the initial values of the parameter optimalization too. According to the concentration curve first-order pharmacokinetic model (‘SSfol’) was used, with the following parameters: initial dose (dose; administered substance in mg), measuring time (hour), elimination rate constant (lKe), absorption rate constant (lKa) and the clearance (lCl). The method was applied using the natural logarithm of the three rates, with all parameters left at their default settings (52). The logarithm of CBD concentration was used as dependent variable and the data were only analyzed up to hour 288, because after that the concentration did not reach the limit of detection. In another model the body mass was used as explanatory parameter instead of the initial dose to explore its relevance in the absorption process. In all models the ID of the individuals was used as random factor. The goodness-of-fit of all models was checked (distribution of residuals, normality of residuals, assess homoscedasticity and detect any sign of nonlinearity).

Data of physical examinations were compared using the heart rate, respiratory rate, bowel movements and auditory stimulation response scoring as dependent variables. There was no variance between the individuals in the touch stimulation response scoring. Horses were divided into two groups based on their age (group “aged” (*n* = 5) more than 6 years and group “young” (*n* = 5) 6 years old or younger). Linear mixed models were built for the heart and respiratory rate, where the independent variables were the following: time of measure (as four level factor), sex and age (two level factor). The ID and the family ID were used as random factor (random intercepts). The time before administration was the reference level. To calculate the exact difference between measurements post-hoc comparisons (with Dunnett contrasts) were used, where all time were compared with the reference level. For bowel movements the median of all-time group was calculated, and these medians and the auditory stimulation response scores were compared with Wilcoxon test (pairwise comparison with the reference level).

A quantile-quantile plot statistical descriptive analysis was performed to assess data normality using the “qqnorm” and “qqline” functions in R (53) in the case of blood hematology and biochemistry parameters before and after administration. If the distribution was normal, they were compared with pairwise t-test. When not, non-parametric test was used, where it was tested whether the median value of the difference between the two measurements is equal to 0 or not. In the case of pairwise t-tests *p*-value correction was used with FDR (54). During analysis “lmer” function in package lme4 (55) was used to build linear mixed models, “nlmer” in package nlme (56) for non-linear mixed models, package multcomp (57) for post-hoc comparison and ggplot2 (58) for plots.

Based on primary results a second CBD peak was noticeable in 5 out of 10 individual plasma concentration–time curves (Supplementary Figure 3). The secondary increase in CBD plasma concentration was between 4 and 6 h after administration. Due to CBD’s lipophilic nature and its tendency to accumulate in adipose tissue, the authors hypothesized that the double peak could be related to the redistribution of CBD, and that the pharmacokinetic variance may stem from differences in the body conditions of the ponies. To investigate this particular feature, a comparison between these five ponies and the others was performed, with particular regard to the body weight and the TG level before and after the CBD administration.

Due to the low sample size, the results should be interpreted with caution. Prior to comparison, normality was assessed graphically using histograms and density plots. The Brunner–Munzel test was applied for body mass, given its non-normal distribution, while Welch’s t-test was used for triglyceride levels.

## Results

### Pharmacokinetics

#### CBD plasma concentration

**Figure 1:**
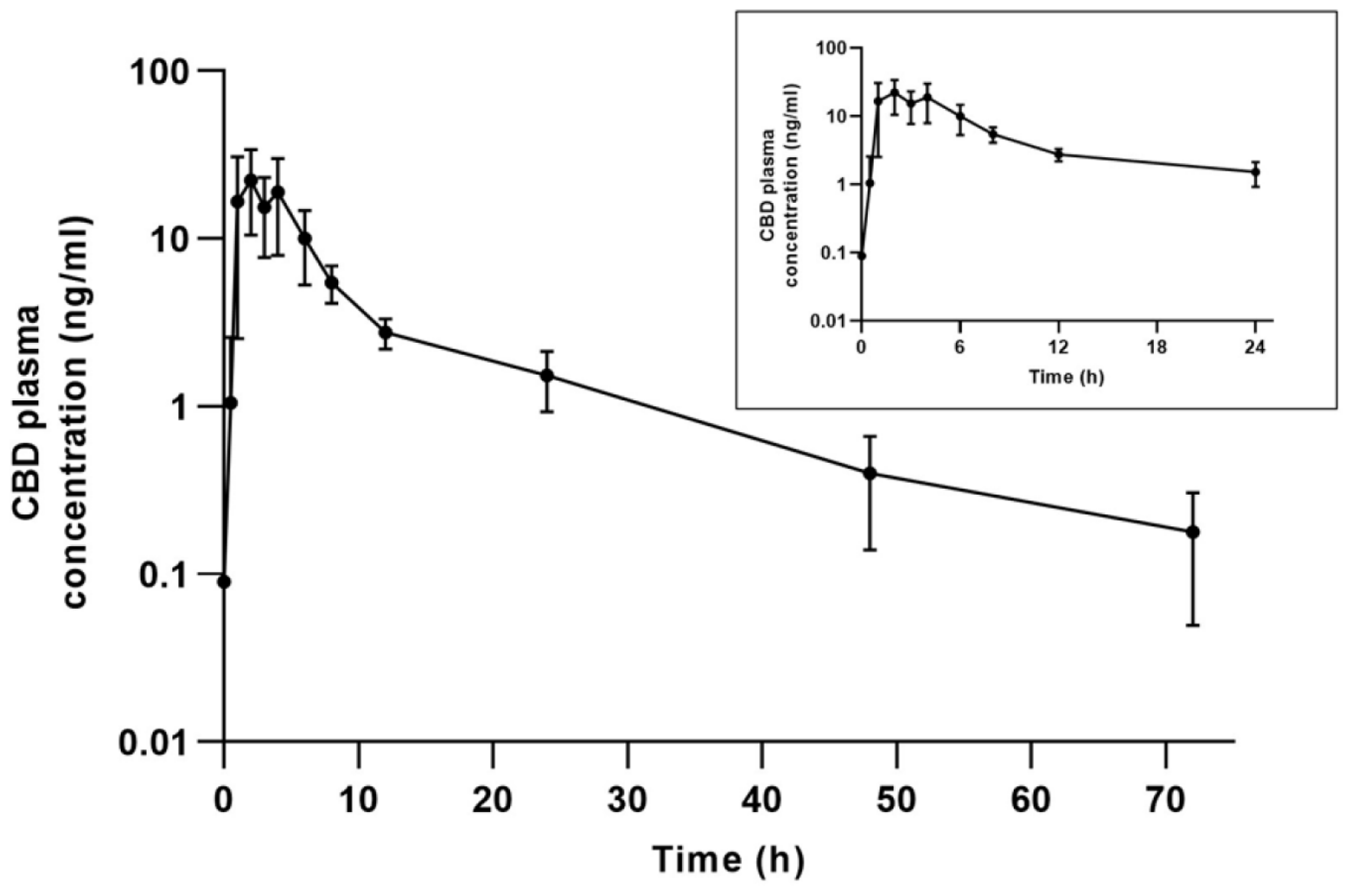
Mean ± SD plasma concentration-time profile for CBD on a semi-logarithmic scale after oral administration of 2 mg/kg CBD to 10 horses.

No cannabinoids were detected in the baseline blood samples. CBD was detected in all subjects at 48 h post-administration, in six subjects at 72 h, and in four subjects at 168 h. Thereafter, CBD was not detectable above the quantitation limit (0.100 ng/mL) in any of the horses (Figure 1).

The mean maximum plasma concentration (C_max_) of CBD and the time to peak concentration (t_max_) were 31.472 ± 8.080 ng/mL and 2.111 ± 0.928 h (Figure 1), while the average half-life (t_1/2_) was 12.563 ± 3.054 h (Table 1). In one aged mare, the CBD concentration reached its maximum 6 h after administration, and it was notably lower (8.740 ng/mL) than average.

**Table 1:** Mean ± SD pharmacokinetic parameters for CBD following a single oral administration of CBD (2 mg/kg) to 10 horses.

Parameter	Unit	Mean	SD	*n*
λ_z_	h^−1^	0.059	0.016	10
t_1/2_	h	12.563	3.054	10
t_max_	h	2.111	0.928	10
C_max_	ng/mL	31.472	8.080	10
AUC _0-t_	ng/mL*h	182.557	43.524	10
AUC _0-inf_	ng/mL*h	186.069	43.838	10
MRT _0-inf_	h	15.868	7.830	10
V_z_/F	(L/kg)	198.757	49.123	10
Cl/F	(L/(kg*h))	11.235	2.396	10

λ_z_, terminal elimination rate constant; t_½_, elimination half-life; t_max_, time of peak plasma concentration; C_max_, peak plasma concentration; AUC _0-t_, area under the plasma drug concentration-time curve from time 0 to 168 h; AUC _0-inf_, area under the plasma drug concentration-time curve from time 0 to infinity; MRT _0-inf_, mean residence time; V_z_/F, apparent volume of distribution; Cl/F, oral clearance.

**Figure 2:**
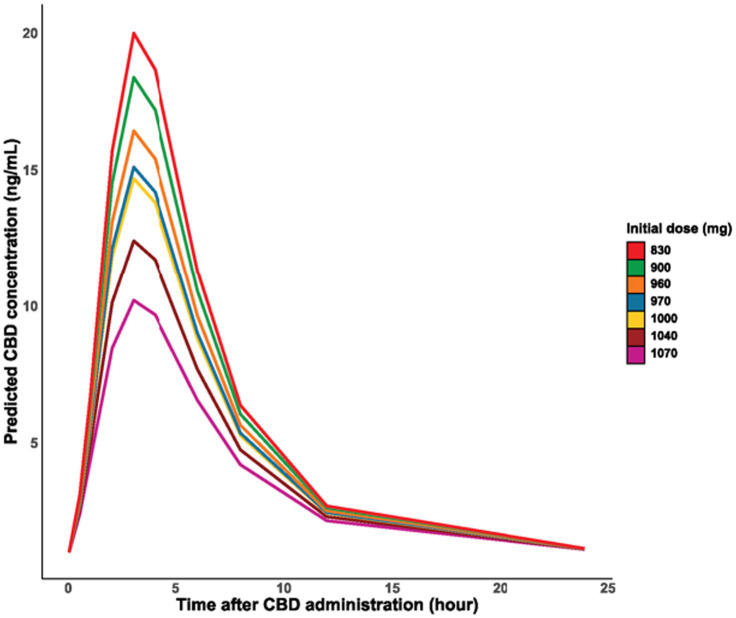
Time course of the logarithm of CBD concentration in blood plasma by dose administered.

**Figure 3:**
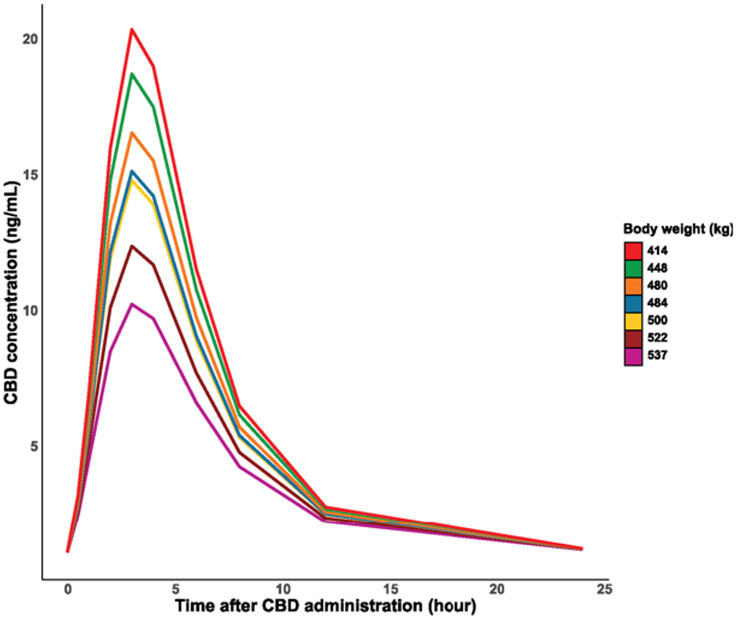
Time course of the logarithm of CBD concentration in blood plasma by body weight of individuals.

According to the models based on the administered dose and body weight, the absorption and final elimination from the body was faster with smaller doses and in smaller body weight (Figures 2, 3). No remarkable variance between individuals could be detected in the models (variance of random effects = 0) (Table 2).

**Table 2:** Non-linear mixed model estimates for CBD constant elimination rate (lKe), constant absorption rate (lKa) and total clearance (lCl).

Parameter	Estimated value ± SD	*t*-value	*p*-value
Model I: Initial dose (mg)			
lK_e_	−0.635 ± 0.706	−0.899	0.369
lK_a_	−1.791 ± 0.691	−2.591	0.010
lCl	−1.052 ± 0.252	−4.167	<0.001
Model II: Body mass (kg)			
lK_e_	−1.790 ± 0.692	−2.588	0.010
lK_a_	−0.635 ± 0.707	−0.899	0.369
lCl	2.861 ± 0.253	11.331	<0.001

The model takes the natural logarithm of all three values, significance levels are from the Anova table.

Individuals with double CBD concentration peak had minimal lower body weight on average (mean±SD, 490.6 ± 43.0 vs. 486.4 ± 20.2 kg; test statistic = −2.889, *p* = 0.004), however, the TG level in blood was significantly higher before the CBD administration, 0.531 mmol/L in the simple peak and 0.731 mmol/L in the double peak group (*t* = −2.518, df. = 13.358, *p* = 0.025). In one aged mare, body weight (448 kg) and TG concentration (1.140 mmol/L) were notably different from the rest of the group, as low body weight was associated with an elevated TG level. When this data point was considered an outlier and excluded from the analysis, the observed differences were no longer statistically significant (body weight mean±SD: 490.600 ± 43.002 vs. 496.000 ± 6.946 kg (test statistic = −0.854, *p* = 0.394) and TG mean±SD: 0.531 vs. 0.644 mmol/L (*t* = −1.8331, df = 13.363, *p*-value = 0.08916)). Of the five individuals in which this double peak was observed, three were younger (aged 5 and 6 years) and two were in the old age groups (aged 19 and 21 years). The data indicates, that four of the five individuals with double peak were female and only one was male.

### Metabolites (7-COOH-CBD, 7-OH-CBD) plasma peak area ratio

**Figure 4:**
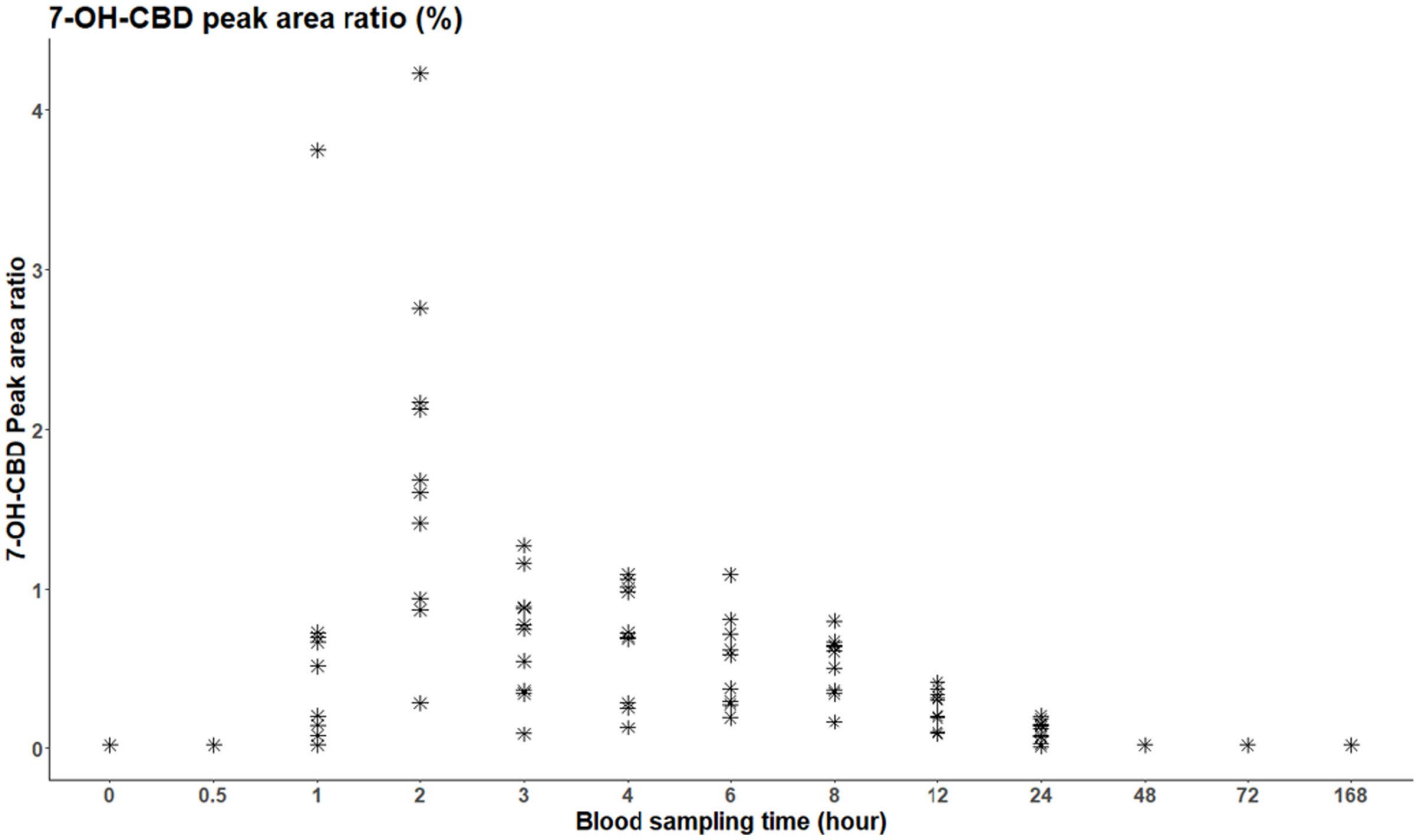
Peak area ratio of 7-OH-CBD in blood plasma per individual; the initial time point (t = 0) represents the time of administration.

**Figure 5:**
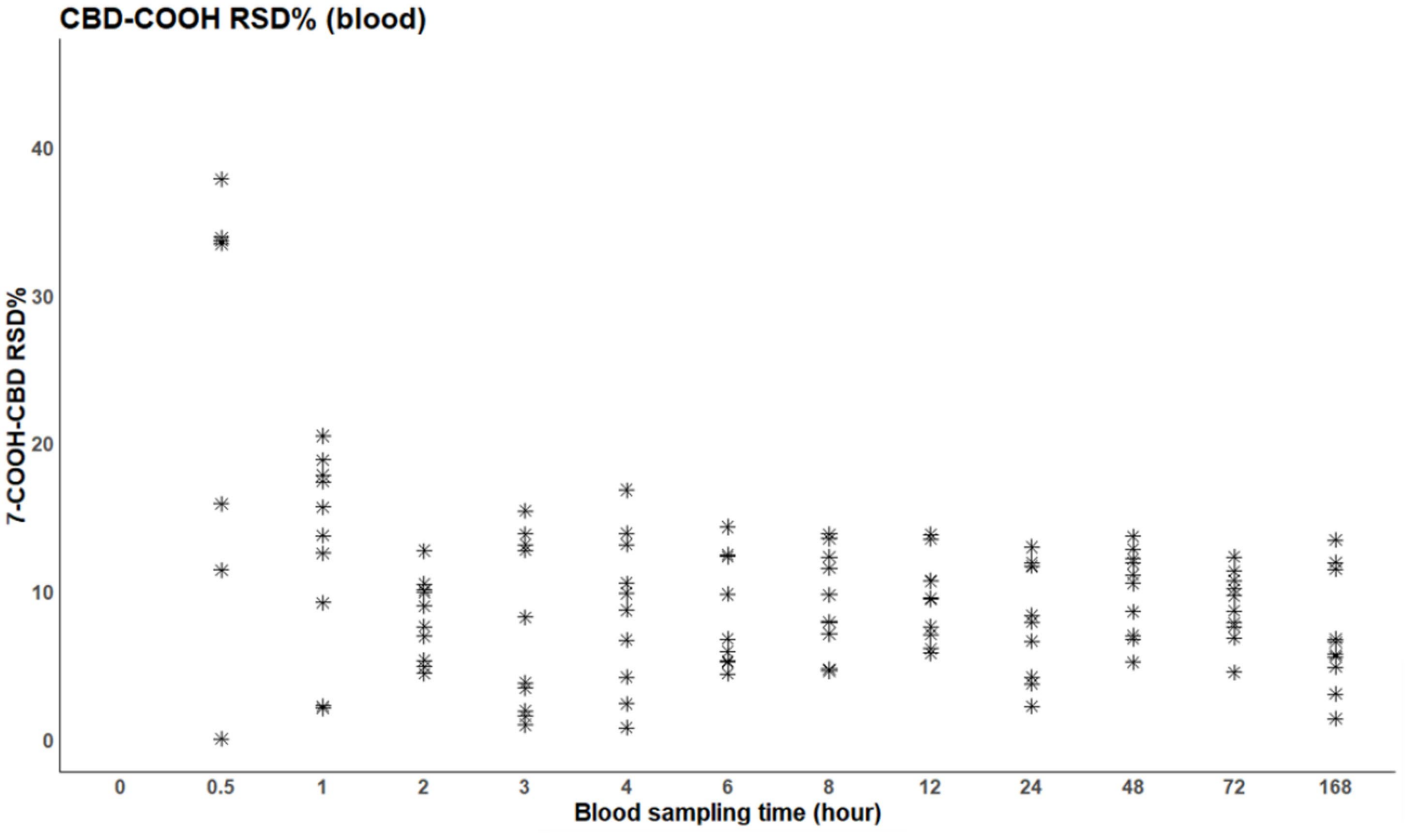
Relative standard deviation of 7-COOH-CBD in blood plasma per individual; the initial time point (t = 0) represents the time of administration.

The 7-OH-CBD metabolite was only present in the plasma between 1 and 24 h after administration (Figure 4). Meanwhile, the dominant metabolite 7-COOH-CBD was already detectable 30 min after the treatment and was above the limit of quantitation in all horses, even 16 days later (Figure 5).

### Vital parameters, side effects

The product was easily administered orally by dosing syringe and was well-tolerated by all horses. No side effects were noted during the experiment. No horse displayed evidence of any degree of discomfort or decreased appetite. All ponies included in the study were found to be clinically healthy. Temperature, pulse rate, and respiratory rate remained within normal limits for all 10 ponies.

**Figure 6:**
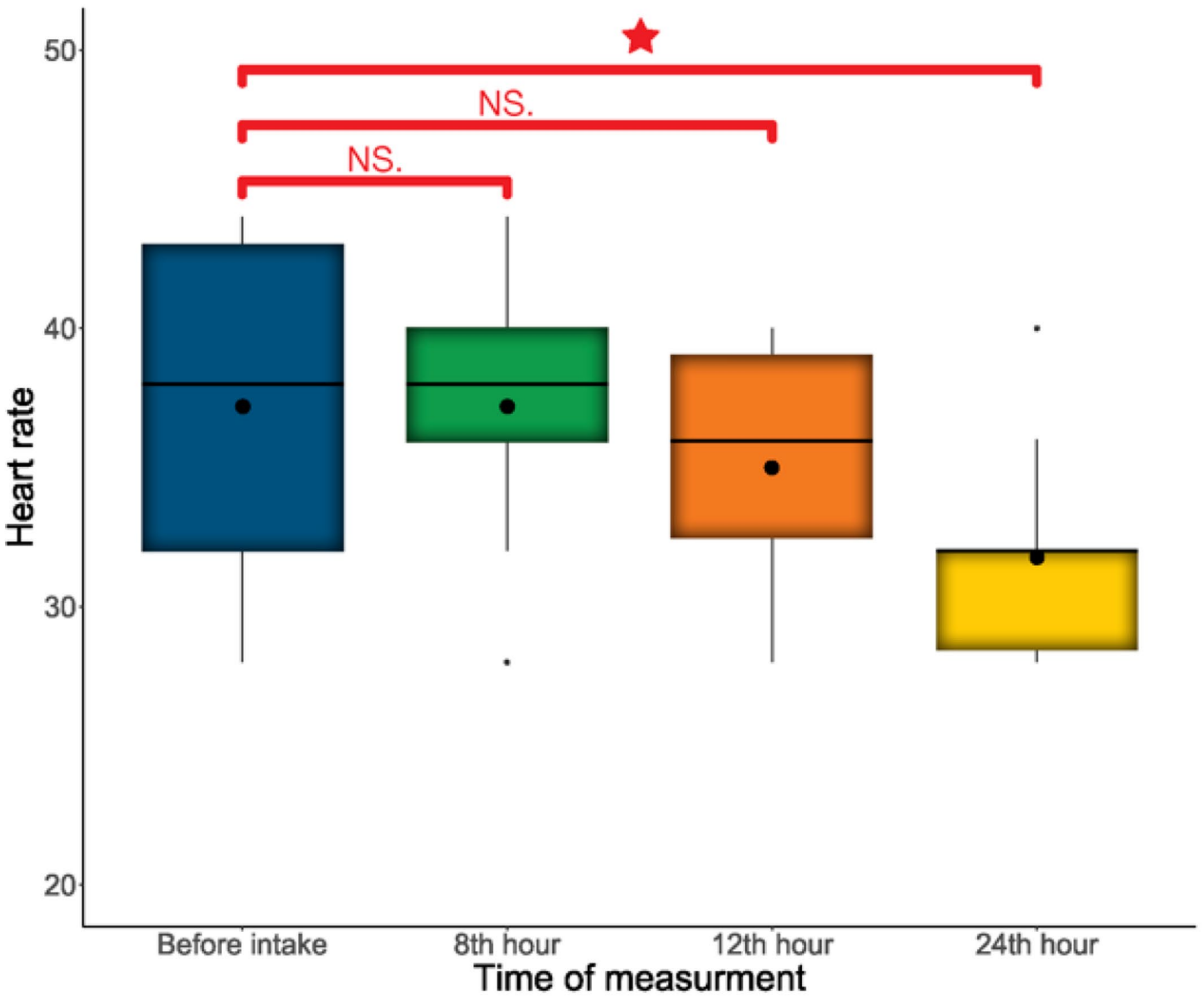
Comparison of heart rate at the four time points. “*” indicates the significant difference.

A significant decrease in heart rate was observed after 24 h of CBD administration based on post-hoc tests (estimate differences±SE = −5.400 ± 1.650, *z*-value = −3.269, *p* = 0.003; Figure 6). There was no similar difference in the respiratory rate (Chi^2^ = 0.485, df = 3, *p* = 0.922), and it did not differ between the two age groups (Chi^2^ = 0.104, df = 1, *p* = 0.747) or between sexes (Chi^2^ = 0.027, df = 1, *p* = 0.867). No difference was detected in temperature, bowel movements, ataxia degree, or the response scores to auditory and touch stimulation.

### Hematology and blood chemistry

Blood hematology and biochemistry analyses were performed before (baseline) and 24 h after administration. At baseline, the RBC count (reference range: 7.8–11 T/L) and HGB levels (reference range: 130–170 g/L) were below the normal range in 7 ponies (RBC: 6.59–7.7 T/L) and in 6 ponies (HGB: 123–129 g/L), respectively. TG levels were also elevated in 9 ponies (0.44–1.14 mmol/L), exceeding the laboratory reference value of <0.4 mmol/*L.* prior to CBD administration, only 4 horses had TG levels above 0.6 mmol/L, with one exhibiting a notably high value of 1.14 mmol/L. LDH levels were elevated in 9 ponies and ranged from 688 to 1,245 U/L (reference range: 80–650 U/L) across all time points. In three horses with LDH levels exceeding 1,000 U/L, CK levels were also slightly elevated, ranging from 229 to 257 U/L (reference range: 20–225 U/L). These parameters did not change significantly following oil administration.

Significant differences were observed in 5 parameters between the two sampling time points (Table 3). However, after *p*-value correction only in the case of urea remained in the acceptable range. Accordingly, significant elevation in the urea (reference range: 3,300–7,400 mmol/L) value (Supplementary Figure 1), slight elevation in the GGT (reference range: 10–45 U/L), TBR (reference range: 9–50 μmol/L), LDH (reference range: 80–650 U/L) values and slight reduction in WBC count (reference range: 5–12 G/L) were observable. The chloride variable (reference range: 89–108 mmol/L) showed a non-normal distribution, so a median test was performed. A significant, however not relevant increase was observed after CBD administration (p-value = 0.008) (Supplementary Figure 2). The average value was before and after CBD administration 100.800 ± 1.476 mmol/L and 101.900 ± 1.853 mmol/L, respectively.

**Table 3:** The mean ± SD values of hematological and other biochemical parameters were calculated from blood taken before and 24 h after CBD administration.

Parameter	Reference range	Before adm.	After adm.	Mean difference	95% CI	*t*-value	*p*-value
WBC	5–12 G/L	6.820 ± 1.064	6.370 ± 0.769	0.450	0.088–0.812	2.812	0.206
GGT	10–45 U/L	14.500 ± 3.895	14.900 ± 4.149	−0.400	−0.769 – −0.031	−2.449	0.206
TBR	9–50 μmol/L	15.630 ± 3.890	16.220 ± 3.797	−0.590	−1.177 – −0.003	−2.272	0.206
LDH	80–650 U/L	897.010 ± 194.899	922.200 ± 199.331	−25.200	−49.028 – −1.372	−2.392	0.206
Urea	3.3–7.4 mmol/L	3.990 ± 0.559	4.410 ± 0.608	−0.420	−0.613 – −0.227	−4.919	0.017

Only those parameters that showed significant differences in the tests before the *p*-value adjustment are shown in the table. The confidence interval (95% CI) refers to the differences between the two measurements, and the adjusted *p*-values were calculated using the FDR method.

## Discussion

During the investigation, the CBD product was easily administered and well tolerated by all ponies, with no side effects observed throughout the experiment. CBD was detected in all subjects 48 h after administration. In 6 ponies, CBD concentrations were below LOQ after 72 h (3 days), and in 4 after 168 h (7 days) (Figure 1). The next sampling occurred on day 12, which is a limitation of the present study as the exact time of CBD elimination from all ponies is unknown. However, CBD was not detectable above the quantitation limit (0.100 ng/mL) in any of the horses on day 12. In the course of the research, the pharmacokinetic analysis showed a rapid increase of CBD in plasma and the mean maximum plasma concentration of CBD was 31.472 ± 8.080 ng/mL following the administration of 2 mg/kg CBD oil. As previously mentioned, commonly used oral CBD doses in horses range from 0.1 to 3 mg/kg, with the most frequently reported maximum serum plasma CBD concentrations being less than 20 ng/mL (32, 36–45). The plasma concentration achieved in the present study is, therefore, considered relatively high. Due to the small amount administered orally with a dosing syringe, oromucosal absorption cannot be ruled out. This, by bypassing first-pass metabolism, may have contributed to the relatively high plasma concentration. The influence of high-fat meals on the intestinal absorption of CBD has been well-documented (59), with absorption enhanced due to its lipophilic nature. Accordingly, a coconut oil-based formulation containing medium-chain triglycerides (MCTs) was used. MCTs, a type of medium-chain fatty acid, do not require bile acids for digestion, allowing them to be quickly absorbed and used as an immediate energy source. This may have contributed to the observed peak plasma concentration in the present study.

The pharmacokinetic profile of CBD is influenced by several factors, including the dose, pharmaceutical formulation, route of administration, the type of use (acute vs. chronic), and the individual characteristics of the patient (15). Previous observations have shown that with oral administration, an increase in dose results in a corresponding rise in maximum plasma concentration (34). Thomson et al. (60) reported a plasma concentration of 40.350 (27.700–52.170) ng/mL after administering 8 mg/kg of CBD via nasogastric tube. In comparison, Sánchez de Medina et al. (3) achieved higher plasma concentrations following the administration of 10 mg/kg CBD orally on an empty stomach. They observed plasma concentrations of 55.660 (35.760–94.340) ng/mL with an oil-based preparation and 142.730 (104.600–354.410) ng/mL with a specific micellar formulation. The C_max_ of approximately 143 ng/mL is notably higher than most reported values, suggesting that using higher doses and specialized formulations, such as micellar preparations, may be effective strategies for enhancing CBD bioavailability and achieving higher plasma concentrations. In another study, a low dose of CBD oil (0.1 mg/kg) was administered transmucosally, resulting in a significant plasma concentration of 27 ng/mL. This effect was likely due to the bypassing of first-pass metabolism in the liver (33). Williams et al. (34) achieved a C_max_ of 51 (± 14) ng/mL by administering a 2 mg/kg dose of full-spectrum CBD pellets once daily for 7 days. The maximal plasma concentration was measured after the final dose, which may contribute to the higher observed levels, as CBD can accumulate in organs and adipose tissue. Additionally, the pellet formulation could have enhanced absorption.

However, even the highest plasma concentrations achieved in horses remain lower than those observed in dogs receiving comparable doses. Deabold et al. (61) reported a maximal plasma concentration of 301 ng/mL following the administration of 2 mg/kg CBD to dogs. The low bioavailability of orally administered medication in horses can be attributed to several factors, including low gastric pH, slow gastrointestinal transit time, the lower fat content of their forage, drug binding to intestinal contents, and the unique digestive enzymes present in horses (44). Interestingly, lower plasma concentrations of CBD have also been observed in guinea pigs. The significantly lower C_max_ in horses and guinea pigs, compared to dogs, may suggest a pronounced first-pass effect. Since both guinea pigs and horses are hindgut fermenters, this indicates that these species may require higher doses of CBD to achieve higher plasma concentrations (45). In humans, the oral bioavailability of CBD is reduced to 13–19% due to first-pass metabolism in the liver and poor solubility (15). In horses, only two studies have reported bioavailability, with values of approximately 8 and 14% (3, 44). Based on studies so far, it appears that the pharmacokinetics of CBD in horses are more similar to humans than dogs (27).

Low CBD plasma concentration does not necessarily indicate inadequate efficacy. It may depend heavily on factors such as species (e.g., receptor distribution) and the desired therapeutic effect. Although the exact mechanism of CBD action remains unclear, receptor localization varies between species. For example, CB1 and CB2 receptors are evenly distributed across the canine epidermis, while in humans, CB1 is found in the spinosum and granulosum layers and CB2 in basal keratinocytes (62). Differences in ligand binding between human and rat CB1/CB2 receptors have also been reported, suggesting that efficacy may vary across species (63). Cannabinoids may exert dose-dependent effects by activating, antagonizing, or inhibiting diverse cellular targets (64). As mentioned in a previous study, a 300 mg oral dose of CBD had anxiolytic effects in humans, while 100 mg and 900 mg were ineffective (46), suggesting that different conditions may require different plasma concentrations. For example, lower doses may alleviate anxiety, while higher doses may be needed for seizure control. In studies where there is a solid rationale for CBD use (e.g., Crohn’s disease and chronic pain), neutral results could be due to subtherapeutic dosing. Therefore, dose-escalation trials with integrated pharmacokinetic studies are the logical next step (65). In horses two case reports described the positive effects of CBD in treating mechanical allodynia and chronic crib-biting and windsucking, using 0.5 mg/kg of CBD administered orally twice a day (28, 29). While these reports did not measure CBD plasma concentrations, previously mentioned studies indicate that maximum CBD concentrations are typically less than 20 ng/mL following oral administration of up to 3 mg/kg. However, as discussed above, the low plasma concentrations do not necessarily preclude effectiveness.

According to the present research, the t_max_ was 2.111 ± 0.928 h, which is consistent with most previous reports. The average time to reach maximum plasma concentration varies from 1–5 h (15). In one aged mare, the CBD concentration peaked 6 h after administration, reaching a notably lower level (8.740 ng/mL) than the average. Significant interindividual variation in both CBD concentrations and concentration-time curves has been reported in previous studies, which could explain the longer t_max_ and the lower maximal plasma concentration (66). The average half-life (t_1/2_) was 12.563 ± 3.054 h, which is significantly longer than in dogs but aligns closely with findings in horses. These findings should be considered when defining dosing regimens and may support a 12-h dosing interval in horses over once-daily administration.

In the present study, the faster absorption and elimination of CBD observed in horses with smaller doses and lower body weight may be related to the accumulation of CBD in adipose tissue. However, body condition scoring, a more accurate indicator of obesity, was not performed. Despite this, the horses’ heights were similar (145–154 cm), suggesting that the variations in body weight were more likely due to differences in body condition rather than height.

The plasma concentration–time curves of THC and its metabolites show a characteristic pattern for oral drug consumption. However, variability was observed between individuals. Similar irregularities were found in the concentration-time curves of CBD and its metabolites (66, 67). In most cases, THC or CBD, after reaching peak plasma concentrations, show a steady decrease. However, in some cases, delayed resorption, intermittent resorption with a shoulder, or a second maximum was observed (66, 67). Similar variability in the resorption kinetics was observed in the present study. A second CBD peak was noticeable in 5 out of 10 animals plasma concentration–time curves. The secondary increase in CBD plasma concentration was between 4 and 6 h after administration. This elevation could be related to the redistribution from fatty tissues.

Cannabinoids are highly lipophilic and lipid-soluble compounds, which leads them to accumulate in fatty tissues and penetrate well-vascularized organs such as adipose tissue, heart, brain, liver, lungs, and spleen (68). A recent research conducted on rats provides clear evidence that CBD accumulates in fat, liver, and muscle tissue. Moreover, at any given dose, the increase in adipose tissue is greater than in muscle or liver (1). This distribution results in a rapid decrease in their plasma concentration (15). After an initial distribution phase, THC redistributes from lipid depots back into blood. CBD metabolism is very similar to THC (68).

To investigate the distinctive pattern observed in the plasma concentration–time curves, a comparison was made between the five ponies exhibiting a double CBD concentration peak and those with a single peak, focusing on body weight and TG levels before and after CBD administration. Ponies with a double peak had slightly lower average body weight compared to those with a single peak (mean±SD, 490.600 ± 43.002 vs. 486.400 ± 20.217 kg; test statistic = −2.889, *p* = 0.004). However, pre-administration TG levels were significantly higher in the double peak group (0.731 mmol/L) than in the single peak group (0.531 mmol/L; *t* = −2.518, df = 13.358, *p* = 0.025). One aged mare showed notably different values—body weight of 448 kg and TG level of 1.140 mmol/L—indicating a potential outlier due to the combination of low body weight and elevated TG. When this individual was excluded, the differences were no longer statistically significant (body weight mean±SD: 490.600 ± 43.002 vs. 496.000 ± 6.946 kg) (test statistic = −0.854, *p* = 0.394) and TG mean±SD: 0.531 vs. 0.644 mmol/L (*t* = −1.833, df = 13.363, *p*-value = 0.089).

Due to the small sample size, this is only an observation, and the long-term relevance remains unclear. In a single-dose pharmacokinetic study conducted on dogs, a prominent increase in CBD and THC plasma concentrations was observed between 12 and 24 h after administration in 5 out of 18 animals. However, this represents a significantly longer period of time compared to the present study. The authors suggested enterohepatic recycling of cannabinoids or coprophagia; however, neither of these theories has been conclusively proven (69).

The main cannabinoid metabolism takes place in the liver. CBD is hydroxylated by CYP450 enzymes to the active metabolite 7-OH-CBD which is further metabolized to the inactive metabolite 7-COOH-CBD (15). The inactive metabolite is substantial in cannabinoid metabolism because it is present in the body for a longer period of time and thus can be a very useful biomarker of cannabis exposure. CBD also undergoes direct glucuronidation via UGTs (9). Cannabinoids mostly excreted in the feces (approx. 65%) and in the urine (approx. 20%) (9, 10). CBD is mainly eliminated unchanged in the feces and as both unchanged and glucuronidated CBD in the urine (9).

The specific metabolite profile can vary significantly between species (42). There is currently no research available that outlines the precise steps of CBD metabolism in horses. In humans, CBD is extensively metabolized, and the rapid increase in metabolite concentrations is due to the extensive first-pass metabolism of the parent compound, which is specific to oral intake (66, 67).

As part of the analysis, the authors expected the inactive metabolite to be detectable in plasma for a longer period. 7-COOH-CBD was above the quantification limit in all horses, even 16 days after a single oral CBD administration. Further investigation is needed to determine the precise elimination time of the 7-COOH-CBD metabolite. The 7-OH-CBD metabolite was only present in the plasma between 1 and 24 h after administration. Eichler et al. (37) explained the low serum plasma value of 7-OH-CBD by the partial metabolism into 7-COOH-CBD. Ryan et al. (42) reported that 7-OH-CBD concentrations remained low in blood throughout the post-administration period, with the carboxylated metabolite being the predominant form. Although urine was not analyzed in the present study, both Ryan et al. (42) and Eichler et al. (37) found that, in equine urine samples, the 7-OH-CBD metabolite was more prevalent than 7-COOH-CBD and CBD itself.

Cannabinoid use is prohibited in human sports competitions, with carboxy-THC serving as the primary marker for cannabis misuse in urine. According to the World Anti-Doping Agency (WADA), concentrations exceeding 180 ng/mL constitute an Adverse Analytical Finding (AAF) (70). An AAF indicates the presence of a prohibited substance or method and may lead to anti-doping rule violations. While no thresholds exist for minor cannabinoids, their detection during in-competition testing may also result in an AAF and subsequent investigation (70). Although CBD was removed from WADA’s Prohibited List in 2018, CBD products derived from cannabis extracts can contain trace amounts of THC or other banned cannabinoids, posing a risk of AAFs if identified in doping controls (70).

Most international equine sport associations list all natural and synthetic cannabinoids as banned substances. However, in 2022, the Fédération Equestre Internationale (FEI) reclassified cannabidiol (CBD) and cannabidiolic acid (CBDA)—the precursor to CBD—as controlled medications and included them among specified substances (37). This change underscores the importance of understanding CBD pharmacokinetics in horses. Detection of a specified substance may still result in an AAF, but such substances are subject to different rules and penalties compared to prohibited substances. This classification accounts for the possibility that the substance may be present due to accidental contamination, incorrect administration, or other factors. Nonetheless, any violation of FEI regulations concerning controlled medications or specified substances can still lead to sanctions, including fines, suspensions, or disqualification, depending on the specific circumstances of the case.

A significant decrease in heart rate was observed 24 h after CBD administration (Figure 1). However, there was no change in the auditory and tactile stimulation response scores, indicating that the response to external stimuli did not reduced. The decrease in heart rate may suggest a calming effect of CBD, but the evidence is low grade, and its clinical relevance remains questionable. Investigating CBD’s calming effect was not a primary objective of the study, but it is an observation worth noting. Further research is warranted, including more detailed evaluations (e.g., reactivity scoring, novel object tests, and heart rate variability).

Hematological and biochemical variations observed in the present study may be attributed to species-specific differences. Lower baseline RBC and HGB levels align with previous findings showing that ponies typically exhibit lower erythrogram values than Thoroughbreds or other horse breeds (71). Elevated baseline triglyceride (TG) levels, seen in 9 subjects, reflect known metabolic differences between ponies and horses, though recent studies challenge the belief that healthy ponies have higher TG levels (71, 72). Elevated LDH levels were noted throughout the study, with concurrent mild CK elevations in some ponies, suggesting potential muscle involvement. Given that ponies tend to have higher lactate levels and LDH is linked to lactate metabolism, proportionally elevated LDH may reflect physiological norms rather than pathology (71, 73, 74).

Table 3 summarizes all hematological and biochemical changes observed between baseline and 24 h, including elevations in urea, chloride, GGT, TBR, and LDH, as well as a slight reduction in WBC count.

Urea is synthesized in the liver and serves as the primary route of nitrogen excretion, making protein catabolism directly related to urea concentrations. Its elimination is mainly renal, as it is freely filtered by the glomeruli and undergoes passive tubular reabsorption. Consequently, reduced tubular flow rates can increase urea reabsorption, thereby elevating blood urea concentrations (75). Dietary protein intake and muscle catabolism have a slight positive effect on urea levels in horses. However, once dietary and metabolic factors are excluded, the primary cause of elevated urea levels is a reduced glomerular filtration rate (75). Urea concentrations may rise earlier than creatinine, particularly in pre-renal azotemia, due to enhanced tubular reabsorption (75).

To the authors knowledge, no definitive evidence links CBD use to adverse renal outcomes, although several studies suggest its potential therapeutic benefits for kidney health. Research on doxorubicin-induced kidney damage in rats showed that CBD reduced oxidative stress, serum creatinine, and inflammatory markers like IL-6 (76). In a mouse model of diabetic nephropathy, CBD hydroxypentylester improved kidney function by reducing creatinine, urea levels, hyperglycemia, apoptosis, and inflammation (77). However, one study indicated that CBD worsened renal damage in streptozotocin-induced diabetic nephropathy, suggesting that CBD may exacerbate nephropathy in type 1 diabetes. While CBD appears safe for kidney function in healthy individuals, caution is advised for those with chronic kidney disease (78).

Elevated urea concentration can be linked to high protein intake or dehydration; however, in the present study, these factors are not relevant. Draeger et al. (32) observed similar changes in blood urea concentrations. Within reference ranges, urea consistently increased with both treatment concentration (they administered 50 mg, 100 mg, or 250 mg CBD to their subjects) and time progression, while creatinine levels did not. They assumed that typically, kidney dysfunction would be indicated by a concurrent rise in both urea and creatinine. They concluded that the administration of each dose in their study caused only minimal changes in blood chemistry concentrations. However, given the slight variations observed in both their study and the present study, it would be prudent to monitor these parameters during prolonged CBD treatment.

Yocom et al. (45) found elevation in liver enzymes (GGT, AST, SDH) while administered sunflower lecithin oil-based CBD at 0.5 mg/kg or 1.5 mg/kg q12 orally for 6 weeks, however the increases were not dose dependent and all values returned to reference intervals or decreased 10 days after the last treatment. In the present study only a slight elevation in GGT, TBR, and LDH values was noted, and none of them were relevant. A study investigated liver safety in healthy adults (*n* = 16) taking therapeutic (1,500 mg) daily doses of CBD for approximately 3.5 weeks. Seven subjects experienced elevated serum alanine aminotransferase (ALT) levels and five exceeded the threshold for drug-induced liver injury. These ALT elevations occurred within 2–4 weeks of CBD initial exposure, but no correlation was found between the elevations and baseline characteristics, CYP2C19 genotype (the primary enzyme involved in phase I metabolism of CBD), or CBD plasma concentrations (79).

Ewing et al. (80) demonstrated that CBD, when orally administered to mice has the potential to cause liver injury. The administration of CBD led to dose-dependent induction of cytochromes and UDP-glucuronosyltransferases, which have been linked to drug metabolism and toxicity. Their 10-day sub-acute study also revealed that CBD doses above 50 mg/kg CBD, while initially tolerated, caused toxicity when repeatedly administered, manifesting as liver injury with elevated liver enzymes (ALT, AST, TBR) and increased liver-to-body weight ratios. Additional research in horses is warranted to assess whether oral CBD administration at higher doses, or over extended durations, could lead to further changes in liver enzymes that might prevent long-term use. The potential effectiveness of CBD or any other co-administered medication may also be affected by the horse’s liver health.

CBD has become widely popular as an alternative or adjunct treatment in both human and veterinary medicine. The authors would like to highlight the pain management related to laminitis among the previously mentioned therapeutic targets of CBD in equine medicine. Equine metabolic syndrome (EMS) is a common underlying cause of laminitis, which is the primary clinical consequence of EMS (81, 82). Additionally, horses suffering from EMS may be at risk for other complications, including hyperlipemia and critical care-associated metabolic derangements such as hyperglycemia and hypertriglyceridemia (81). Ponies are more prone to develop EMS than standardbreds. It has been demonstrated that baseline insulin levels are correlated with height. A similar correlation has been observed in humans, where shorter individuals are at greater risk of developing metabolic diseases (82). In ponies, the correlation between height and several biochemical parameters showed a negative relationship with insulin, glucose, adiponectin, and ACTH levels, and a positive relationship with triglyceride and leptin concentrations (82). Ponies have higher plasma lipoprotein concentrations than standardbreds and appear to be more prone to developing hyperlipidemia when in a negative energy balance (83). Given these differences in carbohydrate and lipid metabolism between ponies and horses, the authors considered it important to study whether these variations could influence the pharmacokinetics of CBD in ponies. To the autohrs knowledge, this is the first study to examine the pharmacokinetic properties of CBD in ponies. The results of the present investigation could serve as a foundation for future pharmacodynamic studies.

The main limitation of the present study was the small sample size (*n* = 10). However, it is important to note that previous research on this topic has also used similar sample sizes (3, 27, 32, 34, 36–45, 60). The alignment of the present findings with existing literature suggests that the sample size employed is acceptable within this context. Another limitation was that only blood, not urine samples were tested. Previous research has shown that cannabinoid metabolism and patient responses can vary significantly, even with comparable doses and formulations—a variability potentially explained by genetic polymorphisms in genes involved in cannabinoid metabolism. Personalized medicine aims to improve drug efficacy and safety, and understanding the pharmacogenomics of cannabinoids is crucial for optimizing dosing, minimizing adverse effects, and avoiding treatment failure. Although pharmacogenomic data related to horses exist in the scientific literature, to the authors’ knowledge, no studies have specifically explored its application to cannabinoids; this remains an important and promising area for future investigation, though it was beyond the scope of the present study (84–86).

## Conclusion

Based on the present study, CBD was detectable in 4 out of 10 ponies on day seven, but in none on day 12. However, the 7-COOH-CBD metabolite remained above the detection limit even on day 16. The basic pharmacokinetic characteristics of CBD in the present study, conducted in ponies, were consistent with findings from previous studies in warmblood horses. Although investigating CBD’s calming effects was not a primary objective, the observed reduction in heart rate—despite unchanged response to external stimuli—may suggest a mild calming effect, warranting further research using more detailed behavioral and physiological assessments. Additional research in horses is warranted to assess whether oral CBD administration at higher doses or over extended durations could lead to changes in liver or kidney function that might limit its long-term use. While effective plasma concentrations of CBD for horses or ponies have not yet been established, the present study provides a foundation for further research to evaluate the therapeutic efficacy of CBD in ponies. These data will inform the design of subsequent trials.

## Data Availability

The raw data supporting the conclusions of this article will be made available by the authors, without undue reservation.

## Glossary

AAF: adverse analytical finding
ALP: alkaline phosphate
ALT: alanine aminotransferase
AST: aspartate aminotransferase
AUC: area under the curve
AUC _0-inf_: area under the plasma drug concentration-time curve from time 0 to infinity
AUC _0-t_: area under the plasma drug concentration-time curve from time 0 to 72 h
BCS: body condition score
CB1: cannabinoid receptors type 1
CB2: cannabinoid receptors type 2
CBC: complete blood count
CBD: cannabidiol
CBDA: cannabidiolic acid
CK: Creatine kinase
Cl/F: oral clearance
C_max_: maximum plasma concentration
CYP450: cytochrome P450
ECS: endocannabinoid system (ECS)
EMS: equine metabolic syndrome
FAAH: fatty acid amide hydrolase
FEI: Fédération Equestre Internationale
GGT: gamma-glutamyl transferase
GLDH: glutamate dehydrogenase
GPR: G protein-coupled receptors
HESI: heated-electrospray ionization
lCl: total clearance
LDH: Lactate dehydrogenase
lKa: absorption rate constant
lKe: elimination rate constant
MCT: medium-chain triglyceride
MRT: mean residence time
nlmer: non-linear mixed models (nlmer)
PPAR: nuclear peroxisome proliferator-activated receptor
RMSSD: root mean square of successive beat-to-beat differences
RPM: revolutions per minute
SDH: Sorbitol dehydrogenase
SDNN: standard deviation of normal-to-normal R-R intervals
t_1/2_: half-life
TBR: total bilirubin
TG: triglyceride
THC: tetrahydrocannabinol
t_max_: time to peak drug concentration
TRP: transient receptor potential channel
UGT: Uridine 5′-diphospho-glucuronosyltransferase
UHPLC-MS/HRMS: ultra-high-performance liquid chromatography-high resolution mass spectrometry
V_z_/F: apparent volume of distribution
WADA: World Anti-Doping Agency
‘SSfol’: first-order pharmacokinetic model
%RSD: relative standard deviation
5-HT: serotonin receptor
7-COOH-CBD: 7-carboxy cannabidiol
7-OH-CBD: 7-hydroxy cannabidiol
λ_z_: terminal elimination rate constant
