# Supplementary figures and images for: Pharmacokinetics of cannabidiol and its two main phase I metabolites in Connemara ponies
Source: Front Vet Sci. 2025 Jun 27;12:1599934. doi: 10.3389/fvets.2025.1599934 (PMC12247600; doi:10.3389/fvets.2025.1599934)

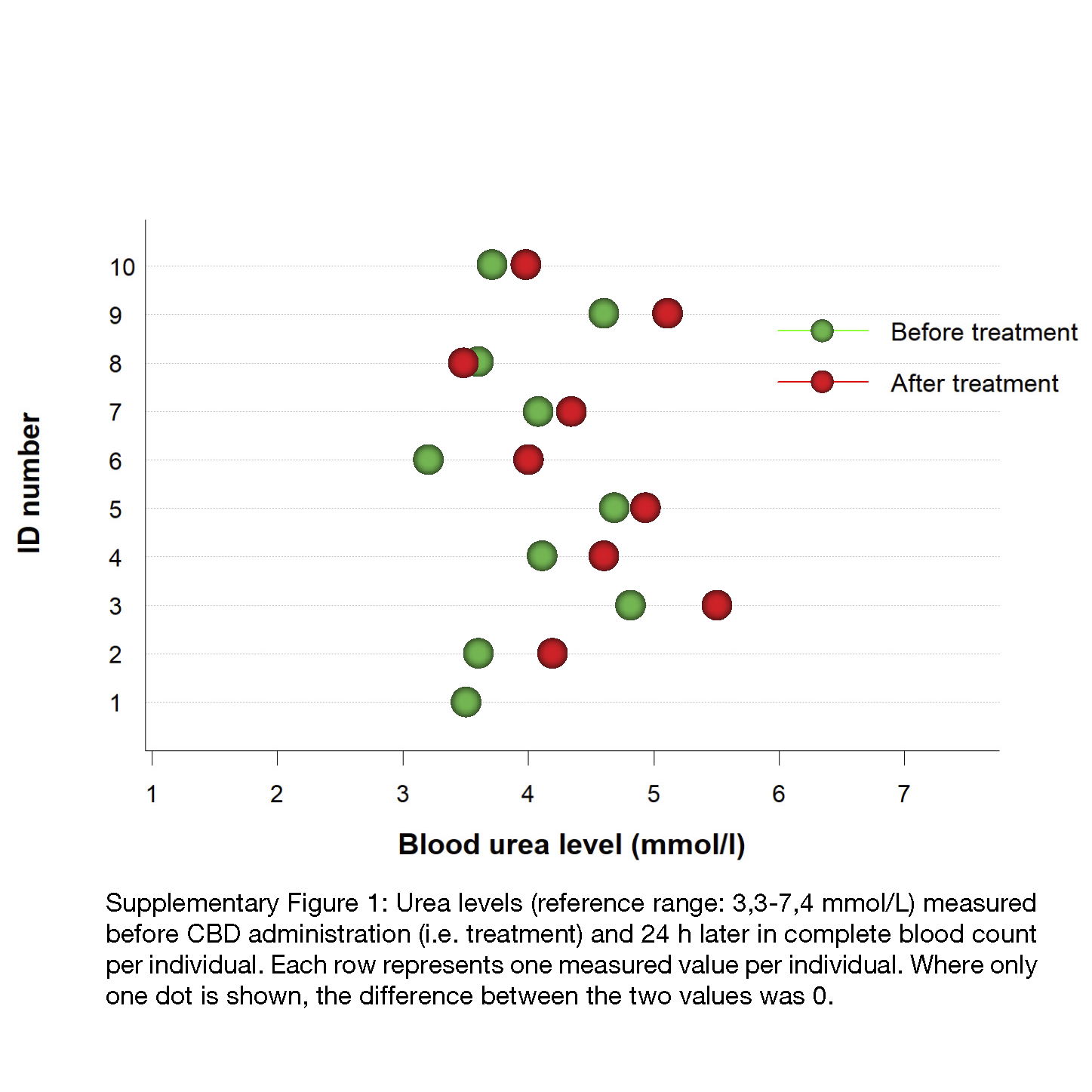

Supplement: Supplementary file 1 [file Image_1.TIFF]

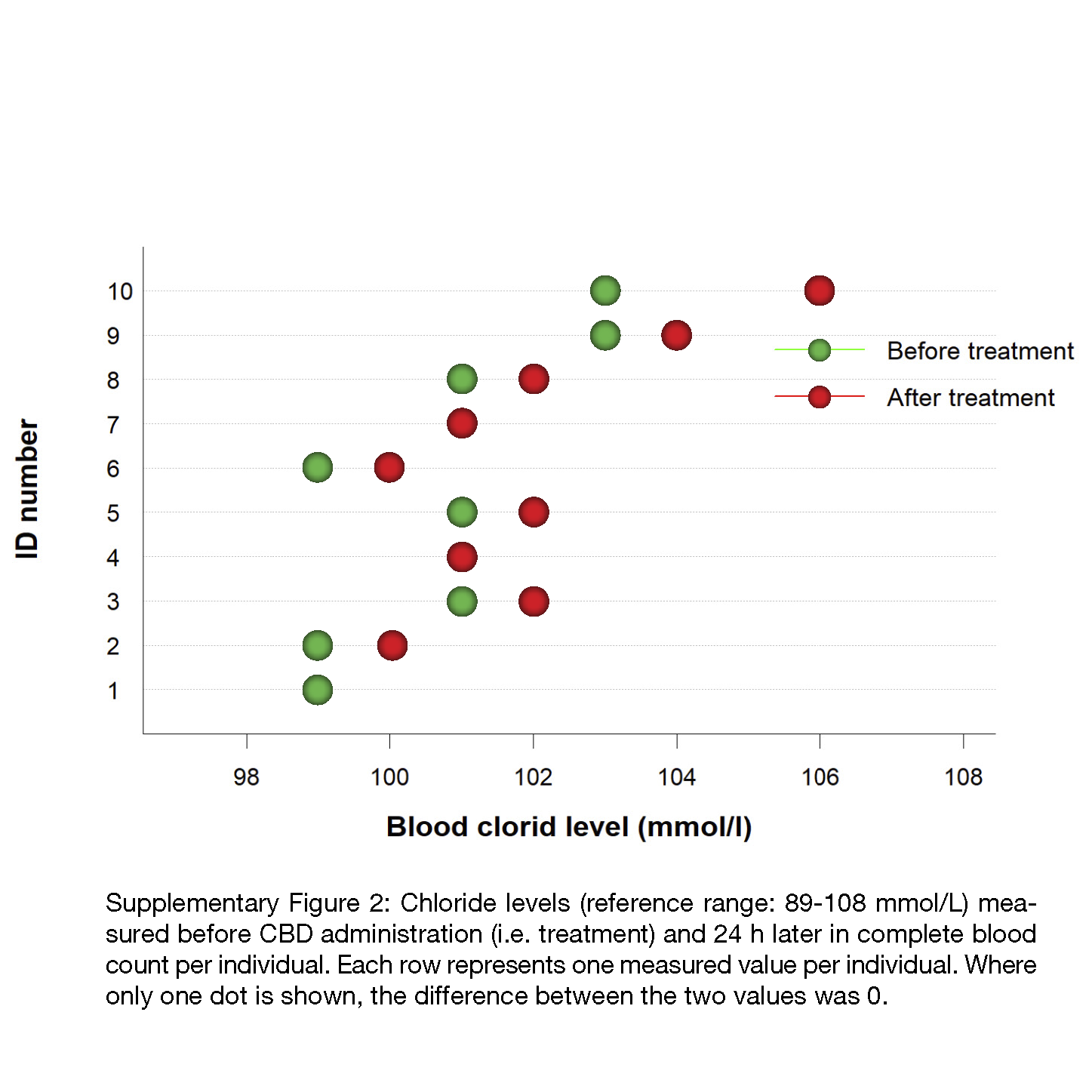

Supplement: Supplementary file 2 [file Image_2.TIFF]

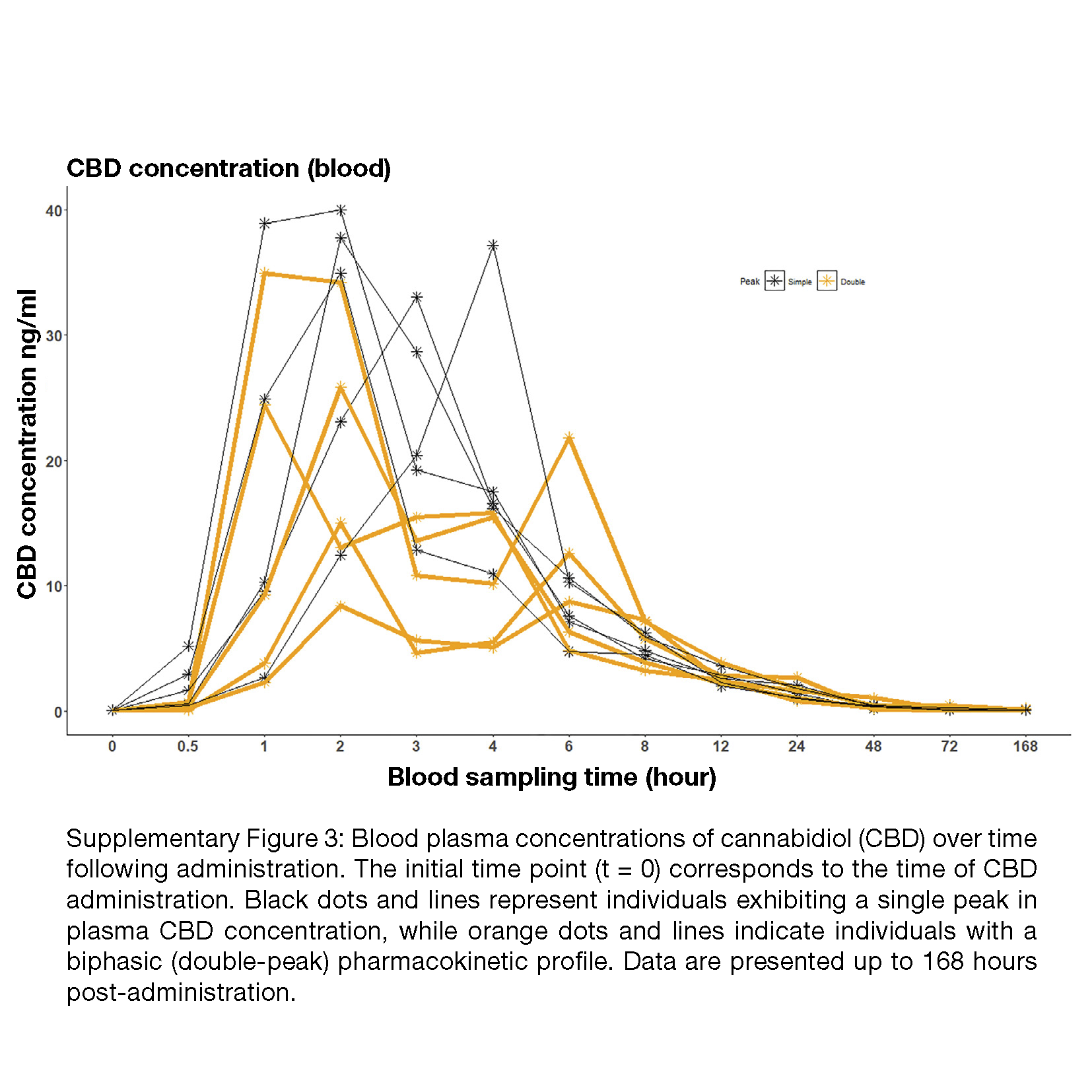

Supplement: Supplementary file 3 [file Image_3.TIFF]
